# Central Odontogenic Myxoma: A Radiographic Analysis

**DOI:** 10.1155/2021/1093412

**Published:** 2021-06-28

**Authors:** Ahmad Badruddin Ghazali, Raweewan Arayasantiparb, Rachai Juengsomjit, Aroonwan Lam-ubol

**Affiliations:** ^1^Department of Oral and Maxillofacial Radiology, Faculty of Dentistry, Mahidol University, Bangkok 10400, Thailand; ^2^Department of Oral and Maxillofacial Pathology, Faculty of Dentistry, Mahidol University, Bangkok 10400, Thailand; ^3^Department of Oral Surgery and Oral Medicine, Faculty of Dentistry, Srinakharinwirot University, Bangkok 10110, Thailand

## Abstract

**Objective:**

This study aimed to determine the radiographic characteristics of odontogenic myxomas (OMs) and their associations.

**Materials and Methods:**

The study enrolled radiographs of patients taken between 2005 and 2019 with a confirmed histopathological diagnosis of central OM. OM radiographic features were evaluated, including location, border, locularity, involved area, the number of included teeth, root resorption, tooth displacement, bone expansion, bone perforation, and periosteal reaction. Fisher's exact test was used for statistical analysis.

**Results:**

Significant associations were found between the OM border and the affected jaw (*p*=0.036), locularity (*p*=0.036), involved areas (*p*=0.009), and bone perforation (*p*=0.036). OMs with an ill-defined border were associated with maxillary lesions, multilocularity, dentate areas, and cortical bone perforation. The number of included teeth (2 or fewer or 3 or more) was significantly associated with locularity (*p*=0.010), involved area (*p*=0.045), and bone expansion (*p*=0.010). Larger OMs including 3 or more teeth, were associated with a multilocular appearance, dentate areas, and bone expansion.

**Conclusion:**

The border of OM and the number of included teeth are related to other radiographic appearances. Understanding these relationships could help in treatment decisions and help better understand the nature of OM.

## 1. Introduction

The World Health Organization classified odontogenic myxoma (OM) as a benign mesenchymal odontogenic tumor in 2017 [1]. It is rare, with a reported annual incidence of 0.07 per million [2], constituting approximately 1.9%–6.3% of all odontogenic tumors [3–5]. The terms myxoma and myxofibroma can be used interchangeably, but when collagen fibers are prominent, the term myxofibroma is appropriate [1, 6]. Although OMs grow slowly, they are invasive and infiltrate the local surrounding bone.

Most OMs are diagnosed in patients during the second to fourth decades of life [7–11]. They are common in the mandible, especially in the posterior region [2, 8, 12, 13]. The OM border in panoramic images can be well-defined (with or without cortication) or ill-defined. OMs exhibit simple to aggressive radiographic manifestations: from a unilocular lesion at the periapical area or surrounding an unerupted tooth [9, 14] to a multilocular lesion mimicking ameloblastoma or odontogenic keratocyst [15], and including a sunburst appearance similar to osteosarcoma [16, 17]. They may displace teeth and cause root resorption. These various radiographic manifestations can lead to an initial misdiagnosis in patients with OM. The current study aimed to evaluate the radiographic characteristics of OM and the interrelationships among them.

## 2. Materials and Methods

The Institutional Review Board, Faculty of Dentistry/Faculty of Pharmacy, Mahidol University reviewed and approved the current cross-sectional study (MU-DT/PY-IRB 2019/025.0205). The inclusion criteria were patients with a confirmed histopathological diagnosis of central OM between January 2005 and December 2019 at the Oral and Maxillofacial Pathology Department, Faculty of Dentistry, Mahidol University. Patients for whom radiographs were unavailable were excluded. A board-certified-oral pathologist (R. J.) reviewed the hematoxylin and eosin-stained sections to confirm the histopathological diagnosis. In total, 12 patients were included in the study.

Panoramic radiographs were used to evaluate radiographic characteristics, including location, border, locularity, involved area, the number of included teeth, root resorption, and tooth displacement. Bone expansion and perforation were evaluated from occlusal cross-sectional, cone beam computed tomography (CBCT), or multidetector computed tomography (MDCT) images, depending on the availability of the imaging modalities for each patient. For evaluation of periosteal reaction, a panoramic radiograph combined with occlusal cross-sectional, CBCT, or MDCT images was used. Panoramic films of three patients were taken with a PM 2002 EC Proline (Planmeca, Helsinki, Finland). Digital panoramic radiographs were taken with a Kodak 9000 C (Carestream Health, Rochester, NY, USA). Ultraspeed or Insight (Eastman Kodak, Rochester, NY) occlusal cross-sectional films were exposed with a GX 1000 (Gendex, IL, USA) or Searcher Dx-068 (Belmont, Osaka, Japan). Occlusal cross-sectional digital radiographs were taken with a Planmeca ProX (Planmeca, Helsinki, Findland) using a phosphor plate system (VistaScan®, Dürr Dental, Bietigheim-Bissingen, Germany). All radiographic films were digitized by scanning with a Microtek ScanMarker 9800XL (Microtek Inc, Santa Fe Spring, CA, USA) with a resolution of 300 dpi. The CBCT imaging was performed with a 3D Accuitomo (J. Morita, Kyoto, Japan) and a CB MercuRay (Hitachi Medical Systems, Tokyo, Japan). One MDCT imaging was performed with a Toshiba Alexion (Toshiba Medical Systems, Tochigi, Japan). All radiographs were evaluated using Picture Archiving and Communication System software installed on a computer running Microsoft Windows 10 (Microsoft Corp., Redmond, WA, USA) and displayed using RadiForce RX430 EIZO (EizoNanao Corporation, Ishikawa, Japan) on a 29.8-inch monitor (2560 × 1600 pixels). All radiographs were examined independently on a computer screen by a board-certified oral and maxillofacial radiologist (R.A.) and a radiologist in training (A. B. G.) in a dimly lit room. The correlation coefficient for the interobserver agreement was 0.99. Any disagreement between the two observers was resolved by consensus.

The location was classified as either anterior (i.e., region from central incisors to canines) or posterior (i.e., region from the first premolar to the tuberosity or ramus). The border was classified into three types: well-defined corticated, well-defined noncorticated, and ill-defined. The border was considered well-defined when an imaginary line could trace the limit of the lesion at the periphery. A corticated border was recorded when a thin, radiopaque line was present at the lesion's periphery. An ill-defined border represented the lesion with an indistinct marginal outline.

Locularity was categorized into unilocular and multilocular. A lesion was considered unilocular when it exhibited a single radiolucent area without any septa; it was considered multilocular when it exhibited at least two compartments with internal septa. Concerning the involved area, lesion presence in dentate, edentulous, or unerupted tooth areas was recorded. The number of included teeth was categorized into 2 types: OM, including two or fewer teeth and three or more teeth. Root resorption and displacement of adjacent teeth were also documented. Bucco-lingual bone expansion, cortical bone perforation, and periosteal reaction were also recorded.

Descriptive statistics were used to describe radiographic features. The Fisher's exact test was used to analyze the association between radiographic features using IBM SPSS Statistics for Windows (Version 21.0., IBM Corp., Armonk, NY, USA). A *p* < 0.05 was considered statistically significant.

## 3. Results

Among the 658 patients with odontogenic tumors who presented at the Department of Oral and Maxillofacial Pathology over the 15 year study period, 15 (2.3%) had OMs, and 13 (2%) had central OMs. The study included 12 patients who had both panoramic images and occlusal cross-sectional, CBCT or MDCT images.

Of the 12 patients, four were men, and eight were women. Patient age ranged between 21 and 51 years (mean age, 32.4 ± 10 years). OMs were found incidentally in the panoramic radiographs of five asymptomatic patients. Six patients presented with the chief complaint of painless swelling in the affected area; the remaining patients complained of gingival abscess in the involved area. Eight patients (66.7%) exhibited a mandibular lesion, while four (33.3%) exhibited a maxillary lesion. Lesions were in the posterior region in eight patients, in the anterior-posterior region in three, and in the anterior region in one. Midline-crossing lesions were found in two patients: one in the maxilla and the other in the mandible.

**Figure 1:**
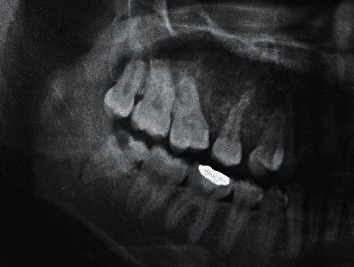
Maxillary odontogenic myxoma in a 26-year-old woman (Patient 3). Cropped panoramic image shows ill-defined multilocular radiolucency located in the dentate area from maxillary right first premolar to the third molar.

**Figure 2:**
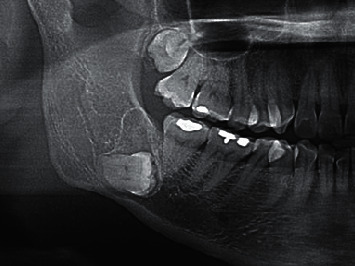
Mandibular odontogenic myxoma in a 44-year-old woman (Patient 9). Cropped panoramic image shows well-defined corticated, unilocular radiolucency associated with unerupted mandibular right third molar.

**Figure 3:**
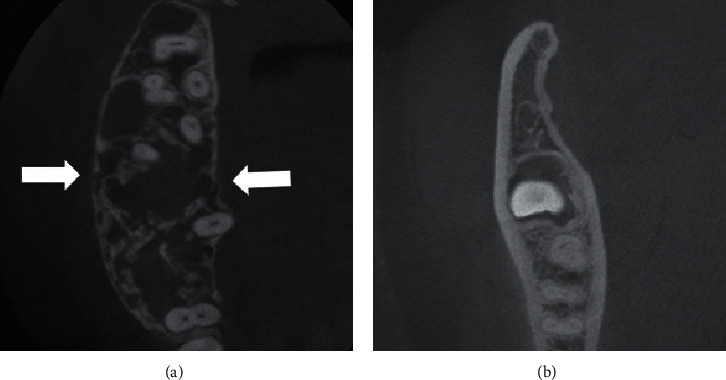
Axial CBCT images. (a) Maxillary odontogenic myxoma (Patient 3) at the dentate area, including more than 3 teeth with bone expansion and perforation (arrows). (b) Mandibular odontogenic myxoma (Patient 9) with unerupted third molar without bone perforation.

The lesion border was well-defined corticated in three patients (25%; all mandibular lesions), well-defined noncorticated in six (50%; five mandibular lesions and one maxillary lesion), and ill-defined in three (25%; all maxillary lesions) (Figure 1). Most OMs (8/12) showed multilocular radiolucency, while four showed unilocular radiolucency. Three unilocular lesions were associated with an unerupted lower third molar (Figure 2). OMs mainly involved the dentate area in nine patients (75%) and unerupted teeth in three (25%—all unilocular OMs). The superior portion of the lesion in all 12 patients was located near the alveolar crest bone. Five OMs (41.7%) included the area of 2 or fewer teeth, and seven OMs (58.3%) included the area of 3 or more teeth. Displacement of adjacent teeth was present in six patients (50%), and root resorption of involved teeth was present in three patients (25%). The bone expansion was found in 8 OMs (66.7%); however, bone perforation was found in 4 OMs (33.3%). All perforation cases had a multilocular appearance, occurred in dentate areas, and had a lesional size, including the area of 3 or more teeth (Figure 3(a)). By comparison, all OMs with unerupted teeth were unilocular with no finding of bone perforation and having a lesional size of OMs including 2 or fewer teeth (Figure 3(b)). There was no periosteal reaction in any of the OMs. The recorded radiographic characteristics are summarized in Tables 1 and 2.

Table 3 shows the relationships between the OM borders and jaws (*p*=0.036), locularity (*p*=0.036), the involved area (*p*=0.009), and bone perforation (*p*=0.036). All OMs with ill-defined borders were maxillary cases, multilocular in appearance, presenting in dentate areas, and having bone perforation. The study also revealed associations between the number of included teeth and locularity (*p*=0.010), involved area (*p*=0.045), and bone expansion (*p*=0.010) in Table 4. All OMs, including area 3 or more teeth, presented multilocularity, occurred in dentate areas, and had bone expansion. There was, however, no association between the OM border and the number of included teeth of OMs. Besides the foregoing, no other association with radiographic features was found.

## 4. Discussion

During the study period, a respective 2.3% and 2% of all patients diagnosed with odontogenic tumors at our institute had OMs and central OMs. Our findings confirm that OMs are rare and are consistent with an OM incidence of approximately 1.9% of all odontogenic tumors in Asians [4, 5]. OM is, however, the second most common odontogenic tumor with a frequency of between 10.3% and 19% of all odontogenic tumors in Africa [18, 19], which might be due to a regional or ethnic difference.

In the current study, OMs occurred more frequently in women (male-to-female ratio of 1 : 2) as in several studies (male-to-female ratio ranging between 1 : 1.8 and 1 : 4.8) [2, 5, 7–10, 12, 20–22]. A male predilection was, however, observed in some studies [13, 23, 24]. Although the age at diagnosis of OM varies, most OMs are observed in patients during the second to fourth decade of life [2, 8–11], including in our study. The peak age of diagnosis was during the third decade of life (50% of patients in the current study), as was found in several previous studies [8–10, 22, 24]. Manila et al. reported that the mean age for male patients (56.7 years) was greater than that of female patients (40.5 years) [23]; by comparison, we found the respective age was 34 and 31.6 years.

Clinical complaints vary among studies. Some studies reported that swelling was the most common clinical complaint of patients with OM [7, 8], while Simon et al. reported that no clinical symptoms were observed in most patients with OM [2]. In the present study, half of the patients presented with the chief complaint of painless swelling in the affected area. Furthermore, 41.7% of lesions were found incidentally on panoramic radiographs taken during routine dental examinations. No patients had pain in our study, whereas pain was present in 25%–28% of patients in some studies [2, 8].

Our findings support the notion that OM is more common in the mandible [2, 7, 8, 22], as was the case for two-thirds of our patients. Some studies, however, found equal incidences of OMs in both the maxilla and mandible [5, 13, 25]. The predominant area of the jaw affected by OM is reportedly the posterior region, especially the premolar-molar region [2, 13] or the posterior mandible and ramus [22, 26]. In our study, two-thirds of our cases exhibited lesions in the posterior region, while three patients (25%) exhibited lesions in the anterior-posterior region. Only one patient exhibited a lesion in the anterior region alone, and this lesion also crossed the midline in the maxilla. These findings agree with the results reported by Takahashi et al., who found that all OMs in the anterior region were maxillary OMs [5]. Of note, we found two midline-crossing lesions (16.7%), both of which were multilocular OMs, one in the maxilla and the other in the mandible. The midline-crossing mandibular lesion was a large OM for which involvement extended from the left premolar region to the right molar region. Similarly, most OMs did not cross the midline in prior reports [5, 27]. A summary of the demographic and radiographic findings of patients in large case series reported in the English-language medical literature in recent decades is shown in Table 5.

In the current study, 25% and 50% of patients had well-defined corticated and noncorticated OM borders, respectively. One-quarter of patients had ill-defined OM borders. Our findings are consistent with previous studies in which well-defined borders were present in between 58% and 84% of patients, while ill-defined borders were present in between 16% and 42% of patients [9, 20, 27]. We found a significant association between the OM border and the affected jaw. Most maxillary lesions had ill-defined borders, while all mandibular lesions had well-defined borders. These findings agree with other studies wherein maxillary OMs are typically ill-defined, while mandibular OMs are typically well-defined [9, 28].

The present study revealed an association between the OM border and locularity. All OMs with an ill-defined border presented multilocularity. All unilocular lesions had well-defined borders. Although OMs can have a unilocular or multilocular presentation, most (66.7%) OMs in the current study were multilocular, as reported by several other studies [2, 8–10, 12, 22, 24, 29]. Takahashi et al. found comparable proportions of unilocular and multilocular lesions—all unilocular lesions were in the maxilla, while all multilocular lesions were in the mandible [5]. While Keszler et al. found that OMs were predominantly unilocular [13], one-third of OMs in our study exhibited unilocular radiolucency—all of which were mandibular OMs.

OMs in our study mainly involved dentate areas in nine patients (75%) and unerupted teeth in three (25%; all unilocular) as with previous reports of a few OM cases found with unerupted teeth [27]. An association between the OM border and the involved area was also observed in our study. All OMs with unerupted teeth had well-defined, corticated borders. An association was also found between the OM border and bone perforation. Most cases with bone perforation had an ill-defined border. All cases without bone perforation had a well-defined border. Although we did not find any previous reports of an association between cortical perforation and OM border, cortical perforation was reportedly associated with large-sized OMs [15]. We were not able to confirm this finding.

OMs including 2 or fewer teeth or 3 or more teeth were significantly associated with the locularity in our study. All unilocular cases were OMs including 2 or fewer teeth, while almost multilocular cases were OMs including 3 or more teeth. Our finding confirms a previous result indicating that OMs with a unilocular appearance tend to include a smaller area than OMs with a multilocular appearance [14, 15, 20, 27]. This observation was supported by Kauke et al. [15], who found that large-sized OMs were associated with multilocularity, so they suggested that multilocularity was one of the signs of aggressiveness in OM. A systematic study in 2020 also found that most of the recurrences reported were multilocular in appearance [12].

OMs including 2 or fewer teeth or 3 or more teeth were also associated with the involved area. All OMs with unerupted teeth were small OMs including 2 or fewer teeth, while most OMs at the dentate area were larger OMs including 3 or more teeth. One previous study reported a large OM with an unerupted tooth [21]. No association has been reported between size and bone expansion or presentation of an unerupted tooth [30]. An association between OMs including 2 or fewer teeth or 3 or more teeth and bone expansion was found in our study. Most expansion cases were large OMs including 3 or more teeth, while all nonexpansion cases were smaller OMs including 2 or fewer teeth.

A notable observation in our study was the superior aspect of all lesions—located near the alveolar crest. Regarding tooth displacement, previous studies found that approximately 20% of patients with OM exhibited tooth displacement [2, 8, 22]. By contrast, the present study found displacement of adjacent teeth in 50% of patients. Root resorption of involved teeth occurred in 25% of patients in the current study, all of whom had mandibular lesions. Previous studies similarly reported that root resorption was present in approximately 20%–50% of patients with OM [2, 5, 22]. As for periosteal reaction, we did not find any cases that showed this radiographic characteristic, although it has been reported as a sunray appearance in some reports [9, 14].

As for imaging evaluation of OM, panoramic radiography and computed tomography are sufficient [12], although Manila et al. suggest that magnetic resonance imaging should also be used to more accurately determine the margins [23]. Panoramic radiography is the most commonly used modality to evaluate OM [12]. Computed tomography gives more detailed information, which is useful for surgical treatment planning that can range from curettage to extensive resection, requiring a multidisciplinary approach for rehabilitation [31].

A limitation of the study was the small number of patients due to the rarity of the tumor. Another limitation was that the 3D volume images were not available for all of the cases. Additional investigations with a larger number of patients and advanced imaging are needed to better understand the nature of the lesion. Further studies to identify the association between 3D images and histopathogenesis—especially using immunohistochemistry—are recommended to better understand the pathogenesis and aggressiveness of some types of lesions.

## 5. Conclusion

OM has a variety of radiographic characteristics. OMs with an ill-defined border were associated with the maxilla, multilocularity, dentate areas, and bone perforation. Large OMs including 3 or more teeth were associated with multilocularity, dentate areas, and bone expansion. There was, however, no association between the OM border and the number of included teeth. Knowing the border and the included teeth (whether few or many) helps to understand the nature of the OM.

## Figures and Tables

**Table 1:** Radiographic features of 12 patients with central odontogenic myxoma—location, border, locularity, involved area, and the number of included teeth.

Patient	Sex	Age	Jaw	Location	Border	Locularity	Involved area	Number of included teeth
1	*F*	34	Maxilla	*R* post	Ill	Multilocular	Dentate area	≥3
2	*M*	31	Maxilla	*L* ant to *L* post	Ill	Multilocular	Dentate area	≥3
3	*F*	26	Maxilla	*R* post	Ill	Multilocular	Dentate area	≥3
4	*M*	32	Maxilla	*R* ant to *L* ant	WDNC	Multilocular	Dentate area	≥3
5	*F*	21	Mandible	*R* post	WDNC	Unilocular	Dentate area	≤2
6	*F*	23	Mandible	*R* post	WDNC	Multilocular	Dentate area	≥3
7	*F*	27	Mandible	*L* post	WDC	Unilocular	Third molar unerupted	≤2
8	*M*	48	Mandible	*R* post *L* post	WDNC	Multilocular	Dentate area	≥3
9	*F*	44	Mandible	*R* post	WDC	Unilocular	Third molar unerupted	≤2
10	*F*	51	Mandible	*L* post	WDC	Unilocular	Third molar unerupted	≤2
11	*F*	27	Mandible	*R* ant to *R* post	WDNC	Multilocular	Dentate area	≥3
12	*M*	25	Mandible	*R* post	WDNC	Multilocular	Dentate area	≤2

*R*: right; *L*: left; Post: posterior; Ant: anterior; WDC: well-defined corticated; WDNC: well-defined noncorticated; Ill: ill-defined.

**Table 2:** Radiographic features of 12 patients with central odontogenic myxoma—root resorption, tooth displacement, bone expansion, bone perforation, and periosteal reaction.

Patient	Sex	Age	Root resorption	Tooth displacement	Bone expansion	Bone perforation	Periosteal reaction
1	*F*	34	No	Yes	Yes	Yes	No
2	*M*	31	No	Yes	Yes	Yes	No
3	*F*	26	No	Yes	Yes	Yes	No
4	*M*	32	No	Yes	Yes	No	No
5	*F*	21	Yes	Yes	No	No	No
6	*F*	23	No	No	Yes	No	No
7	*F*	27	No	No	Yes	No	No
8	*M*	48	Yes	Yes	Yes	Yes	No
9	*F*	44	No	No	No	No	No
10	*F*	51	No	No	No	No	No
11	*F*	27	Yes	No	Yes	No	No
12	*M*	25	No	No	No	No	No

**Table 3:** Relationships of odontogenic myxoma borders with jaws, locularity, involved area, bone perforation, bone expansion, number of included teeth, root resorption, and tooth displacement.

	Border (*n* = 12)			*p* value
	WDC	WDNC	Ill	
Jaw				
Maxilla	0	1	3	0.036^*∗*^
Mandible	3	5	0	
Locularity				
Unilocular	3	1	0	0.036^*∗*^
Multilocular	0	5	3	
Involved area				
Unerupted tooth	3	0	0	0.009^*∗*^
Dentate area	0	6	3	
Bone perforation				
Yes	0	1	3	0.036^*∗*^
No	3	5	0	
Bone expansion				
Yes	1	4	3	0.267
No	2	2	0	
Number of included teeth				
2 or fewer teeth	3	2	0	0.053
3 or more teeth	0	4	3	
Root resorption				
Yes	0	3	0	0.345
No	3	3	3	
Tooth displacement				
Yes	0	3	3	0.123
No	3	3	0	

WDC: well-defined corticated; WDNC: well-defined noncorticated; Ill: ill-defined. ^*∗*^Significant relationship (*p* < 0.05).

**Table 4:** Relationships between the number of included teeth of odontogenic myxoma and jaw, locularity, involved area, bone perforation, bone expansion, border, root resorption, and tooth displacement.

	Included teeth (*n* = 12)		*p* value
	≤2 teeth	≥3 teeth	
Jaw			
Maxilla	0	4	0.081
Mandible	5	3	
Locularity			
Unilocular	4	0	0.010^*∗*^
Multilocular	1	7	
Involved area			
Unerupted tooth	3	0	0.045^*∗*^
Dentate area	2	7	
Bone perforation			
Yes	0	4	0.081
No	5	3	
Bone expansion			
Yes	1	7	0.010^*∗*^
No	4	0	
Border			
Well-defined corticated	3	0	0.053
Well-defined noncorticated	2	4	
Ill	0	3	
Root resorption			
Yes	1	2	1.000
No	4	5	
Tooth displacement			
Yes	1	5	0.242
No	4	2	

^*∗*^Significant relationship (*p* < 0.05).

**Table 5:** Demographic and radiographic characteristics of patients with central odontogenic myxoma in large case series (>10 patients) reported since 2000 in the English-language medical literature.

Author and year	Population	Patients	Sex M : F	Age (years)	Jaw	Location	Border	Locularity	Unerupted teeth	Perforation	Resorp	Displace
					Max : Mand	*A* : *P* : *A* + *P*	Well: Ill	Uni : Multi				
Banasser et al. [7], 2020	USA	38	13 : 25	6–84	15 : 23	7 : 31 : 0	NS	8 : 11	NS	NS	NS	6
								19 NS				

Takahashi et al. [5], 2018	Japan	12	4 : 8	27–65	6 : 6	6 : 6:0	10 : 2	6 : 6	NS	7	4	NS

Vasconcelos et al. [32], 2018	Brazil	85	40 : 45	10–61	44 : 39	26 : 42	NS	17 : 14	NS	NS	NS	NS
					2 NS	17 NS		54 NS				

Francisco et al. [33], 2017	Brazil	14	3 : 11	7–51	3 : 11	NS	NS	5 : 9	NS	NS	NS	NS

Wang et al. [14], 2017	China	18	6 : 12	6–75	6 : 12	NS	14 : 4	3 : 15	2	15	8	12

Titinchi et al. [20], 2016	South Africa	29	8 : 21	7–44	11 : 18	0 : 21 : 5	25 : 4	8 : 18	NS	NS	1	20
						3 NS		3 NS				

Etemad-Moghadam et al. [34], 2014	Iran	40	17 : 23	NS	14 : 26	1 : 36 : 3	NS	NS	NS	NS	1	1

Kheir et al. [30], 2013	South Africa	33	10 : 23	NS	16 : 17	NS	NS	17 : 13	3	6	3	21
								3 NS				

Friedrich et al. [21], 2012	Germany	14	3 : 11	8–45	5 : 9	4 : 7:3	8 : 6	9 : 4	2	NS	2	8
								1 NS				

Martinez-Mata et al. [8], 2008	Mexico, Guatemala, Brazil	62	19 : 43	9–71	25 : 37	13 : 38 : 11	NS	23 : 39	NS	NS	NS	NS

Zhang et al. [9], 2007	China	41	22 : 19	4–63	17 : 24	0 : 22 : 17	24 : 17	7 : 12	NS	2	10	21
						2 NS		22 other characteristics				

Noffke et al. [10], 2007	South Africa	30	9 : 21	NS	11 : 19	1 : 24 : 5	11 : 17	6 : 24	3	7	13	22
							2 NS					

Li et al. [24], 2006	China	25	13 : 12	6–66	13 : 12	4 : 19 : 2	12 : 10	1 : 22	11	NS	3	11
							3 NS	2 NS				

Simon et al. [2], 2004	Tanzania	33	12 : 21	0–64	8 : 24	7 : 20 : 5	NS	4 : 16	NS	7	11	NS
					1 NS	1 NS		13 NS				

Koseki et al. [28], 2003	Japan	17	8 : 9	10–66	8 : 9	0 : 16 : 1	9 : 8	9 : 8	NS	9	8	11

This study	Thailand	12	4 : 8	21–51	4 : 8	1 : 8:3	9 : 3	4 : 8	3	4	3	6

M: male; F: female; Max: maxilla; Mand: mandible; *A*: anterior; *P*: posterior; *A* + *P*: anterior and posterior; Well: well-defined; Ill: ill-defined; Uni: unilocular; Multi: multilocular; Resorp: root resorption; Displace: displacement of teeth; NS: not specified.

## Data Availability

The research data used to support this study are included within the article.
